# Neuroinflammatory Response to Postnatal Administration of Valproic Acid in Wistar Rats as a Mechanism for the Development of Autism Spectrum Disorders: The Role of Neutrophils

**DOI:** 10.3390/brainsci16080831

**Published:** 2026-08-05

**Authors:** Anna Stakhanova, Svetlana Zozulya, Natalya Kost, Olga Voskresenskaya, Anastasiya Pozdnyakova, Elena Cheremnykh, Yulia Chaika, Ekaterina Semina

**Affiliations:** 1 Federal State Budgetary Scientific Institution “Russian Mental Health Research Center”, Moscow 115522, Russia; anna.stahanova@inbox.ru (A.S.); nat-kost@yandex.ru (N.K.); fanianastya@gmail.com (A.P.); elcher10@yandex.ru (E.C.); berseneva76@yandex.ru (Y.C.); e-semina@yandex.ru (E.S.); 2 Faculty of Biology, Lomonosov Moscow State University, Moscow 119234, Russia; voskresenskaya05@mail.ru

**Keywords:** postnatal valproate model of autism spectrum disorders, rats, social behavior, neuroinflammation, neutrophil, neutrophil elastase, complement system, αlpha1-proteinase inhibitor, blood serum, cerebellum

## Abstract

**Highlights:**

**What are the main findings?**

- Postnatal administration of VPA induces an autism-like phenotype in rats.
- Autism-like behavior is associated with increased activity of neutrophil elastase, α1-proteinase inhibitor, and the complement system, indicating systemic inflammation.

**What are the implications of the main findings?**

- Increased activity of neutrophil elastase in the cerebellum indirectly indicates neutrophil infiltration into the central nervous system.
- Neutrophils are suggested to be involved in the neuroinflammatory mechanism of autism spectrum disorders.

**Abstract:**

**Background/Objectives**: According to current concepts, neuroinflammation is one of the putative causes of autism spectrum disorders (ASD) development. However, the role of neutrophils in neuroinflammation remains insufficiently studied. The study was aimed to determine the role of neutrophils in the neuroinflammatory mechanism of ASD development based on a comparative analysis of physiological and behavioral disturbances and the inflammatory response to early postnatal administration of valproic acid (VPA) to Wistar rats. **Methods**: The study was performed on 38 rat pups of both sexes, half of which were injected intraperitoneally with an aqueous solution of VPA at a dose of 150 mg/kg from 6 to 12 postnatal days (PND); control rats received water. Standard physiological and behavioral tests were used: weight monitoring, pain sensitivity (Hot Plate test) on 25 PND, and social behavior (sib/non-sib test) on 55 PND. Neutrophil elastase (NE) and alpha1-proteinase inhibitor (α1-PI) activity in serum and cerebellum homogenate was measured spectrophotometrically. Complement system (CS) activity was analyzed by the death rate of *Tetrahymena pyriformis* ciliates in the presence of rat serum. **Results**: Early postnatal administration of VPA to Wistar rats induces physiological and behavioral changes characteristic of ASD, confirming the validity of the experimental model used. These changes are accompanied by increased activity of inflammatory factors (CS, α1-PI, NE) in the rat serum, indicating the development of inflammation. VPA treatment increased NE activity in the cerebellum, which may indicate neutrophil infiltration of the brain and neuroinflammation development. **Conclusions**: The data obtained indicate the role of neutrophils in neuroinflammatory mechanisms of ASD development.

## 1. Introduction

Autism spectrum disorders (ASD) are developmental disorders that cause social, communication, and behavioral problems. In the middle of the last century, autism was a relatively rare condition. But over time, more and more children began to suffer from this disorder. Statistics show that the incidence of ASD in children over the past 30–40 years in countries where such statistics are collected has increased from 4–5 to 50–116 cases per 10,000 children. Traditionally, ASD are considered more prevalent in males, with an estimated prevalence ratio of approximately 4:1 based on population-based studies. However, evidence accumulated in recent years suggests that this difference may be partly due to diagnostic bias: girls and women with ASD are more likely to exhibit behavioral camouflaging—the use of compensatory and masking strategies to conceal autistic features in social situations, leading to underdiagnosis and delayed recognition of the disorder. Women diagnosed with ASD have been found to subjectively exhibit more pronounced camouflaging and assimilation strategies than men, despite comparable levels of autistic traits, further complicating clinical assessment [1].

Currently, there is no unified concept for the pathogenesis of ASD. Some progress has been made in studying the genetic basis of ASD, but the exact genes have not yet been identified. The nature of the observed changes suggests that the disorders underlying ASD are caused by abnormal development of the nervous system [2,3,4,5]. It is suggested that the development of ASD may be triggered by pre- and perinatal immune dysregulation caused by genetic vulnerability, infectious agents, maternal immune dysfunction during pregnancy, birth trauma, etc. [6,7,8]. The development of inflammatory and autoimmune reactions may also be a consequence of impaired intestinal barrier function [9,10].

According to current concepts, neuroinflammation is one of the putative causes of ASD development [2,10,11,12,13,14]. Immunohistochemical studies have revealed altered expression of a number of cytokines in the brain of patients with ASD [15,16,17]. A prolonged inflammatory process in the nervous tissue can cause serious disturbances leading to neuronal death, impaired myelination, and anatomical and biochemical changes in brain structures [4,5,18]. Signs of inflammatory reactions are also detected in the periphery, as evidenced by increased levels of proinflammatory cytokines (TNF-α, IL-1β, IL-6) in the serum and cerebrospinal fluid of patients with autism [12,19,20]. In addition, there is an increase in the level of acute-phase proteins (C-reactive protein), changes in the kallikrein-kinin system, the complement system, enzymes responsible for vascular permeability, as well as autoantibodies to antigens of nervous tissue in the blood of patients with ASD [21,22,23,24,25,26]. This increase is not found in all patients with ASD, but is characteristic primarily of the most severe forms [12,23]. Thus, activation of the immune system with an increase in a number of inflammatory and autoimmune markers is predominantly associated with psychotic forms of ASD [27].

Among the inflammatory markers measured in the blood and used to assess the level of inflammation, neutrophil elastase (NE), a proteolytic enzyme that characterizes neutrophil activation during the inflammatory response, is well-known. Our previous study reported increased serum leukocyte elastase (the same as NE) in children with psychotic forms of ASD and associations with selected clinical measures, indirectly reflecting the severity of the pathological process in the brain [27].

To determine whether blood parameters correlate with what’s happening in the brain, animal studies using experimental models of ASD are needed. When developing these models, it is important to consider that the early neonatal period is a crucial stage in the life of humans and animals, during which the central nervous system undergoes intensive development, rendering this system significantly vulnerable to environmental factors. Various pharmacological and stressful influences have a negative impact in early life. The consequences of such influences can cause mental illness. Environmental factors that may increase the risk of developing ASD include pharmacological medications during pregnancy, older parental age, preterm birth, low birth weight, complications during pregnancy and childbirth, inflammation, and neonatal stress [28,29,30,31]. For example, taking VPA as an antiepileptic drug during pregnancy significantly increases the risk of developing ASD in the child [32]. This phenomenon has been termed fetal valproate syndrome and has become the basis for developing guidelines limiting the use of VPA in women of reproductive age and for identifying safer alternatives. On the other hand, VPA is used to model ASD in animals. VPA acts through several mechanisms. It inhibits histone deacetylase, which is involved in altering chromatin structure and thus regulates the expression of various genes [32]. For example, decreased expression of the glutamate decarboxylase gene (an enzyme that catalyzes the synthesis of GABA from glutamate) can affect the balance of excitatory/inhibitory processes in the nervous system [33]. Furthermore, VPA stimulates the Wnt signaling pathway, which is involved in regulating the differentiation of neurons in the cerebral cortex. Prenatal and early postnatal administration of VPA to rats was found to cause disorders similar to fetal valproate syndrome, accompanied by morphological changes in brain development, which, in turn, can impact behavior and cognitive functions [34,35,36]. VPA-exposed offspring have been shown to exhibit reduced social interaction and social play, increased stereotypic and repetitive behaviors, and increased anxiety-like behavior in standard tests such as the open field and elevated plus maze [35,36,37,38,39]. These changes largely reflect the core behavioral manifestations of ASD in humans and are therefore considered an important argument in favor of the translational validity of the VPA model. Decreased pain sensitivity is also characteristic for both patients with ASD and in the VPA experimental model of this disease [36,40,41,42].

Several studies have shown that in rats exposed prenatally to VPA, behavioral and neurobiological changes are sex-specific: males are more often described as having more severe impairments in social interaction, while females exhibit more pronounced anxiety-like reactions and stereotypical behavior, as well as differences in neurochemical and neuroinflammatory parameters [43]. These data highlight the need to explicitly consider animal sex when interpreting behavioral phenotypes and planning future studies aimed at comparing sex-specific mechanisms in preclinical models and in patient populations.

The aim of the study was to determine the role of neutrophils in the neuroinflammatory mechanism of ASD development based on a comparative analysis of data on physiological and behavioral disturbances and the inflammatory response to early postnatal administration of valproic acid (VPA) to Wistar rats.

## 2. Materials and Methods

### 2.1. Declarations

All experimental and investigational procedures were compliant with the recommendations of the Guide for the Care and Use of Laboratory Animals, published by the US National Institutes of Health (Publication No. 85-23, revised 2011), and were approved by the Bioethics Commission of Moscow State University (Institutional Review Board Statement: Reg.No. 12.3 from 17 November 2022). Along the experimental period, every attempt was made to limit the animals’ suffering.

### 2.2. Animals

The experimental study was carried out using thirty-eight Wistar rat pups, whose parents were obtained from the Stolbovaya nursery (Russia; http://www.pitomniksto.ru (accessed on 10 May 2022)). The pups (18 males and 20 females) were born in 3 litters. Each litter was randomly divided into 2 groups: control animals (8 males and 10 females) received intraperitoneal injections of water, while experimental animals (10 males and 10 females) received an aqueous solution of VPA (sodium valproate, Sigma, St. Louis, MO, USA) at a dose of 150 mg/kg daily during the period from the 6th to the 12th postnatal days (PND) of life. Rats were housed under standard vivarium conditions, with free (ad libitum) access to water and food and a 12 h light/dark cycle (9:00 AM–9:00 PM). All animal experiments were performed between 10:00 AM and 8:00 PM.

The researchers who conducted the behavioral experiments and biochemical studies were blinded to the allocation of animals and samples to specific experimental groups. Although the experiment used animals of both sexes, sex difference analysis was not performed due to the small sample size.

**Figure 1:**
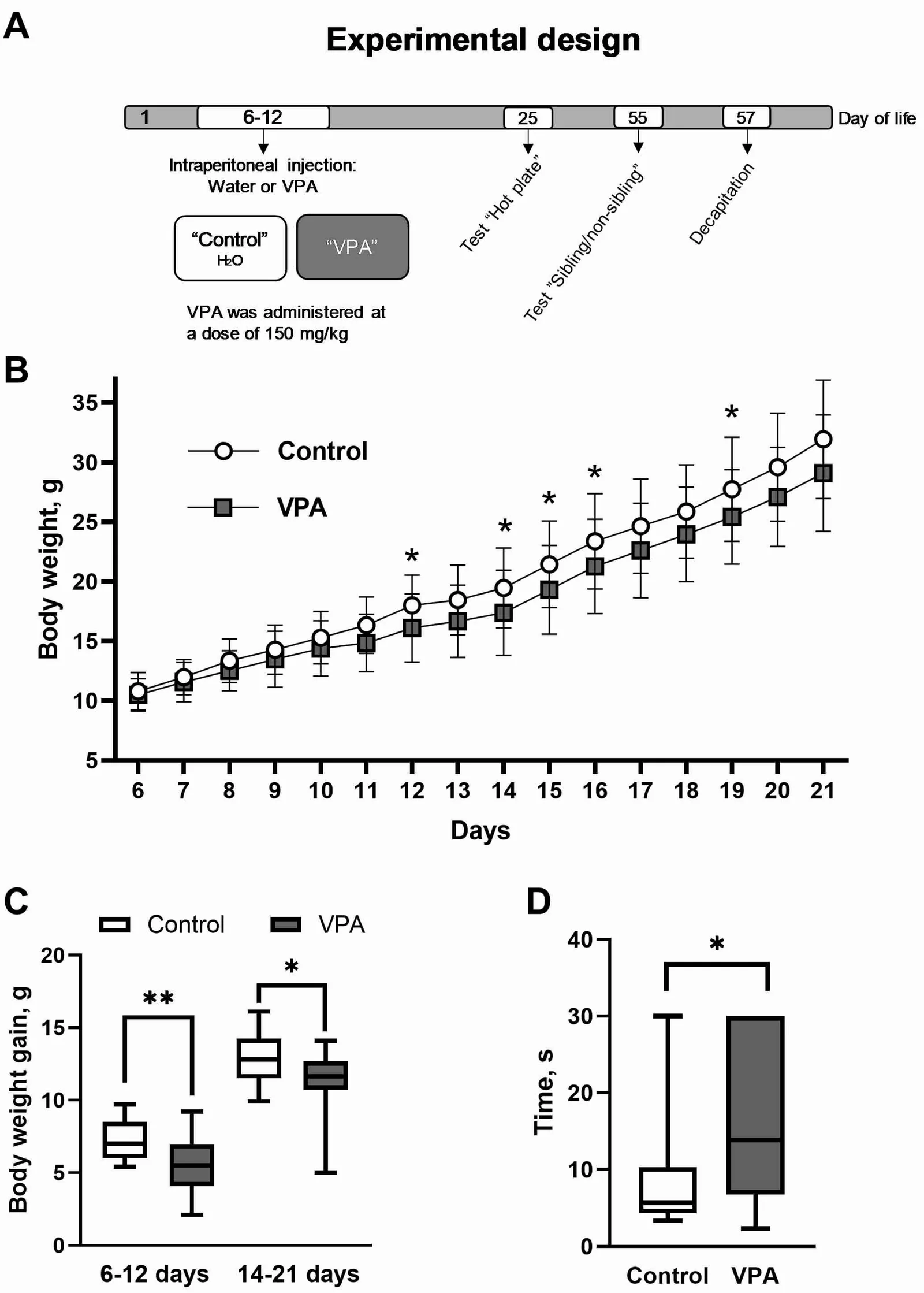
**The effect of postnatal administration of valproic acid (VPA) on physiological development of rat pups.** (**A**) Experimental design. Control animals (8 males and 10 females) received intraperitoneal injections of water, while experimental animals (10 males and 10 females) received an aqueous solution of VPA at a dose of 150 mg/kg daily during the period from the 6th to the 12th postnatal days (PND) of life. Pain sensitivity was detected in the Hot Plate test at 25 PND. Social behavior was tested at 55 PND. Biological materials were obtained at 57 PND. (**B**) Weight monitoring was performed during the first three weeks of life. The weight of animals that received VPA was lower at 12, 14, 15 and 16 PND (* *p* < 0.05). (**C**) As a result, a decrease in weight gain in rat pups received VPA was discovered both for 6–12 (** *p* < 0.01) and 14–21 (* *p* < 0.05) PND. (**D**) Hot Plate test. The pain threshold was recorded as the period from the time when the pups were put on the plate heated to 55 °C to the time that they licked their feet. It was longer in experimental group than in control one (* *p* < 0.05).

The study used standard physiological and behavioral tests: weight monitoring during the first three weeks of life, pain sensitivity assessment on 25 PND, and “Social Behavior” on 55 PND (Figure 1A).

### 2.3. Hot Plate Test

The pups at 25 PND were placed in the test chamber, where the temperature of the heating plate was controlled at 55 °C. The pain threshold was recorded as the period from the time when the pups were put in to the time that they licked their feet.

### 2.4. Social Behavior Test (Sib/Non-Sib Modification) (55 PND)

**Figure 2:**
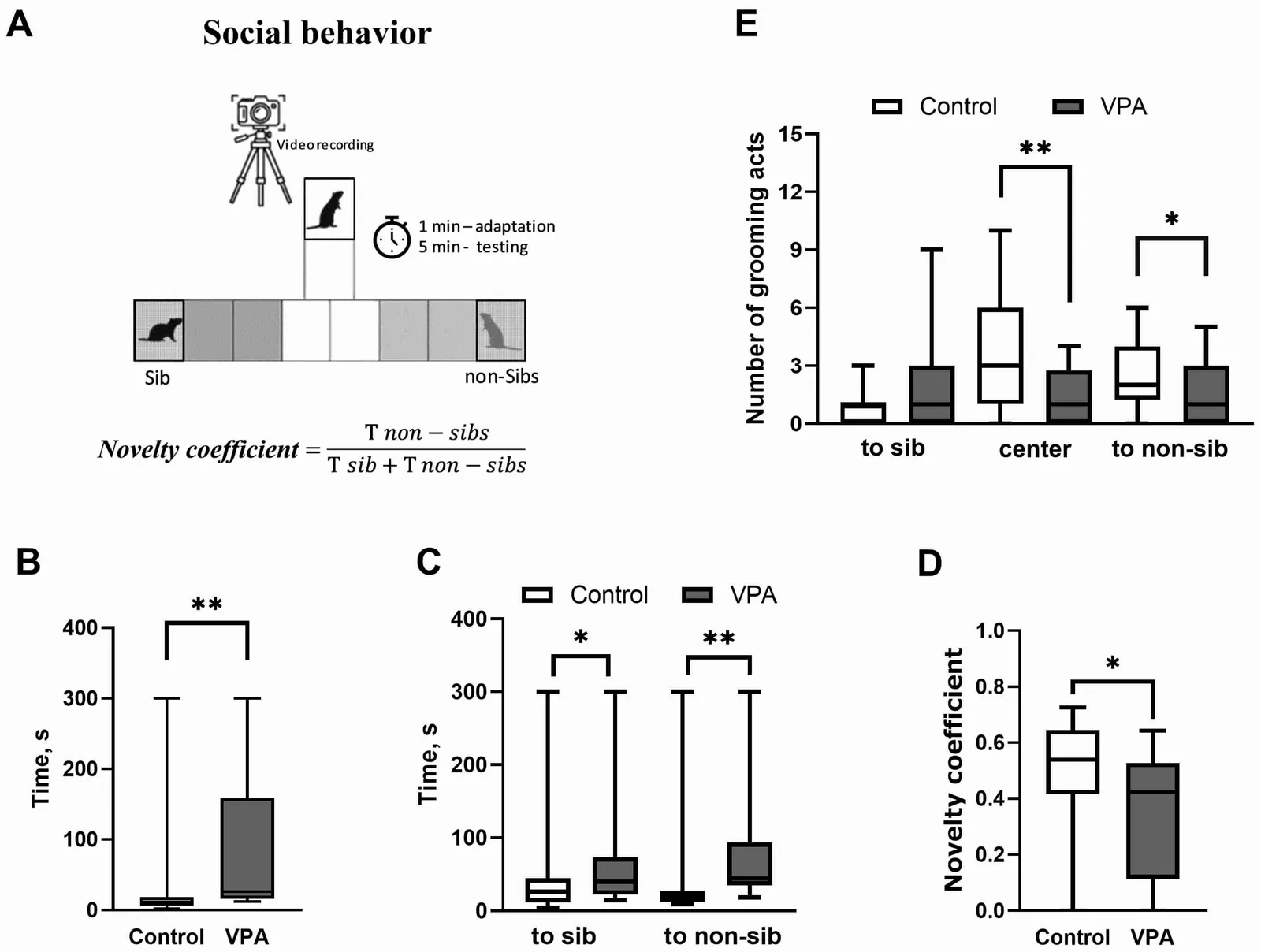
**The effect of postnatal administration of valproic acid (VPA) on the social behavior of animals**. (**A**) Design of the three-chamber test apparatus for rats. The apparatus contained three parts: two side chambers and one central space. The right-side chamber housed non-sibling rat (non-sib), sibling rat (sib) was placed in the left one. The subject rat was placed in the starting section separated by a partition for one minute for adaptation. After partition removing the test rat allowed to freely interact with the social objects. The novelty coefficient was calculated as relation between the time spent in the square near non-sib and the total time spent near sib and non-sib. (**B**) Animals treated with VPA showed significantly increased latency to exit the starting compartment to central space (** *p*< 0.01). (**C**) The latent period of approaching to both sib (* *p* < 0.05) and non-sib (** *p* < 0.001) was longer in VPA treated group compared to control. (**D**) Impaired social behavior of animals received VPA was evidenced by decreased novelty coefficient (* *p* < 0.05). (**E**) One of the indicators of the emotional state of animals is grooming. Decreased number of grooming acts was detected in VPA treated group in the central squares (** *p* < 0.01) and in the non-sib area (* *p*< 0.05). Control animals (8 males and 10 females) received intraperitoneal injections of water, while experimental animals (10 males and 10 females) received an aqueous solution of VPA at a dose of 150 mg/kg daily during the period from the 6th to the 12th postnatal days (PND) of life.

Rats were tested on the 55 PND in a T-shaped maze consisting of squares: 6 in the main section and 2 in the starting section (Figure 2A). Each square measures 14 × 14 cm, with walls 30 cm high. Before testing, rats were placed in the side sections of the maze behind the bars to serve as the objects of social interaction: siblings (sib) and non-siblings (non-sib). The study took place under a red lamp. The subject rat was placed in the starting section of the maze and separated by a partition for one minute—the adaptation period. For 5 min after the partition was removed, the following parameters were recorded: the latent period of exiting the starting compartment; the distance traveled is the total number of squares that the rat passed in 5 min; rearing is vertical motor activity; grooming is touching the animal’s muzzle with the forepaws; the latent period (of entering the side compartments) of approaching the side compartments; the number of approaches, contacts (acts of sniffing), climbs onto the grid, as well as the time (T) spent by the studied animal in the square near the objects of social interaction, from which the novelty coefficient was calculated using the formula.

### 2.5. Biological Material

On 57 PND, the animals were rapidly decapitated, trunk blood was collected into serum separator gel tubes (BD Vacutainer^®^ SST™II Advance), centrifuged at 2000× *g*, and the serum was frozen and stored at −78 °C.

The brain was removed immediately after decapitation and dissected in the cold. Brain sections, in particular the cerebellum, were weighed, frozen in liquid nitrogen and stored at −78 °C. On the day of testing, brain tissue was homogenized in cold 50 mM Tris-HCl buffer pH 7.2 with 0.32 M sucrose (1:5) and centrifuged first at 1000× *g* for 15 min, 4 °C, and then the supernatant was ultracentrifuged (Optima L-90, Beckman Coulter, Brea, CA, USA) at 60,000× *g* for 60 min, 4 °C. The protein concentration in the supernatant and in the blood serum was determined according to Lowry (Bio-Rad DC Protein Assay, Hercules, CA, USA).

### 2.6. Neutrophil Elastase (NE) Activity Assay

The activity of NE was determined by the rate of hydrolysis of N-tert-butoxycarbonyl-alanine-β-nitrophenyl ester (N-t-BOC-Ala-p-NP, BOC) (Sigma, St. Louis, MO, USA) as substrate [44]. The substrate was pre-dissolved in acetonitrile and added to the incubation medium at a concentration of 0.2 mM. Incubation was carried out at room temperature in 50 mM PBS, pH 6.5. The concentration of the resulting product (nitrophenol) was measured spectrophotometrically at 347 nm. The reaction rate was determined from the initial linear interval of the kinetic curve and expressed as U = μM/min.

### 2.7. α1-. Protease Inhibitor (α1-PI) Activity Assay

The inhibitory activity of α1-PI in test samples was determined by inhibition of the BAEE-esterase activity of trypsin [44]. Trypsin activity (AT) was determined by the rate of hydrolysis of BAEE (N-α-benzoyl-L-arginine ethyl ester hydrochloride (ICN Biomedical Inc., Costa Mesa, CA, USA)). The concentration of the resulting product was measured at 253 nm using spectrophotometer Ultrospec 1100 (Amersham, Buckinghamshire, UK). The reaction rate was determined from the initial portion of the kinetic curve. The reaction mixture contained PBS pH 6.5, 50 mM BAEE and 0.5 mg/mL trypsin, pre-incubated with test samples for 3 min. The inhibition units (U) were determined by the difference between AT and AT with sample.

### 2.8. Complement System (CS) Assay

The functional activity of CS in the blood serum was determined in vitro using protozoan unicellular microorganisms-free-living freshwater ciliates *Tetrahymena pyriformis*. The cell death rate was assessed in a 30 mM TRIS-HCl buffer, pH 7.5, containing 2.5 mM CaCl_2_, 1.5 mM MgCl_2_, 50 mM NaCl, and 1.25% serum [45]. The number of active ciliates was counted using a Biolat device designed for cyclic counting of motile organisms and the AutoCiliata image processing computer program for 60 min or until their complete death in the reaction mixture [46]. CS activity is inversely proportional to the immobilization time of half (T50) of the initial number of ciliates, which was 10,000–15,000 active cells/mL.

### 2.9. Statistical Analysis

Statistical processing of the results was performed using statistical programs “Statistica 10.0”, “GraphPadPrism 8.0”. The first stage of the analysis was testing the data for normal distribution using the Shapiro–Wilk test. Normally distributed data were compared using a parametric criterion (Student’s *t*-test), while distributions other than normal were analyzed using the nonparametric Mann–Whitney test and Spearman’s rank correlation coefficient (R). The Benjamini–Hochberg procedure was applied for multiple testing corrections.

## 3. Results

### 3.1. The Effect of Postnatal Administration of Valproic Acid (VPA) on the Physiological Development of Rat Pups

Monitoring the weight of rats from 6 to 21 days of postnatal development (PND) showed that on 12, 14, 15 and 16 PND, the weight of animals that received VPA was lower than in the control (Figure 1B). As a result, a decrease in weight gain in rats of the experimental group was discovered both for 6–12 (*p* = 0.0042) and 14–21 (*p* = 0.0335) PND (Figure 1C). According to the literature, a slowdown in weight gain in the early postnatal period is considered a physiological manifestation of ASD in this model [36].

Decreased pain sensitivity is also characteristic of ASD [36,40,41,42]. This study showed that on 25 PND, rats treated with VPA had an increased paw-licking latency in the Hot Plate test compared to the control (*p* = 0.038) (Figure 1D).

Thus, the observed physiological disturbances caused by postnatal administration of VPA confirm the validity of the experimental model used.

### 3.2. The Effect of Postnatal Administration of Valproic Acid (VPA) on the Social Behavior of Animals

The main indicator of ASD is impaired social behavior. In the Social Behavior test, animals treated with VPA showed significantly increased latency to exit the starting compartment (*p* = 0.0016) and approach time to both siblings (*p* = 0.029) and non-siblings (*p* = 0.00006) compared to controls (Figure 2B,C). The disruption of social behavior of animals that received VPA is also evidenced by a relative decrease in the time spent with a non-sib, as a result of which the coefficient of social novelty in this group was lower than in the control (*p* = 0.03) (Figure 2D).

Affective disturbances are often observed in ASD [37]. One of the indicators of the emotional state of animals is grooming. The Social Behavior test showed that VPA -treated rats had decreased total grooming (8.7 ± 1.1 and 5.3 ± 0.9 in the control and experimental groups, respectively, *p* = 0.024), grooming in the central squares of the installation (4.7 ± 1 and 1.6 ± 0.4 in the control and experimental groups, respectively, *p* = 0.003) and in the area of contact with a non-sib (3 ± 0.4 and 1.5 ± 0.4 in the control and experimental groups, respectively, *p* = 0.016) (Figure 2E).

Thus, the analysis of social behavior of animals on 55 PND showed that early postnatal administration of VPA causes behavioral disturbances characteristic of ASD in pre-adulthood.

### 3.3. The Effect of Postnatal Administration of Valproic Acid (VPA) on the Activity of Inflammatory Factors in the Blood Serum and Cerebellum of Rats

**Figure 3:**
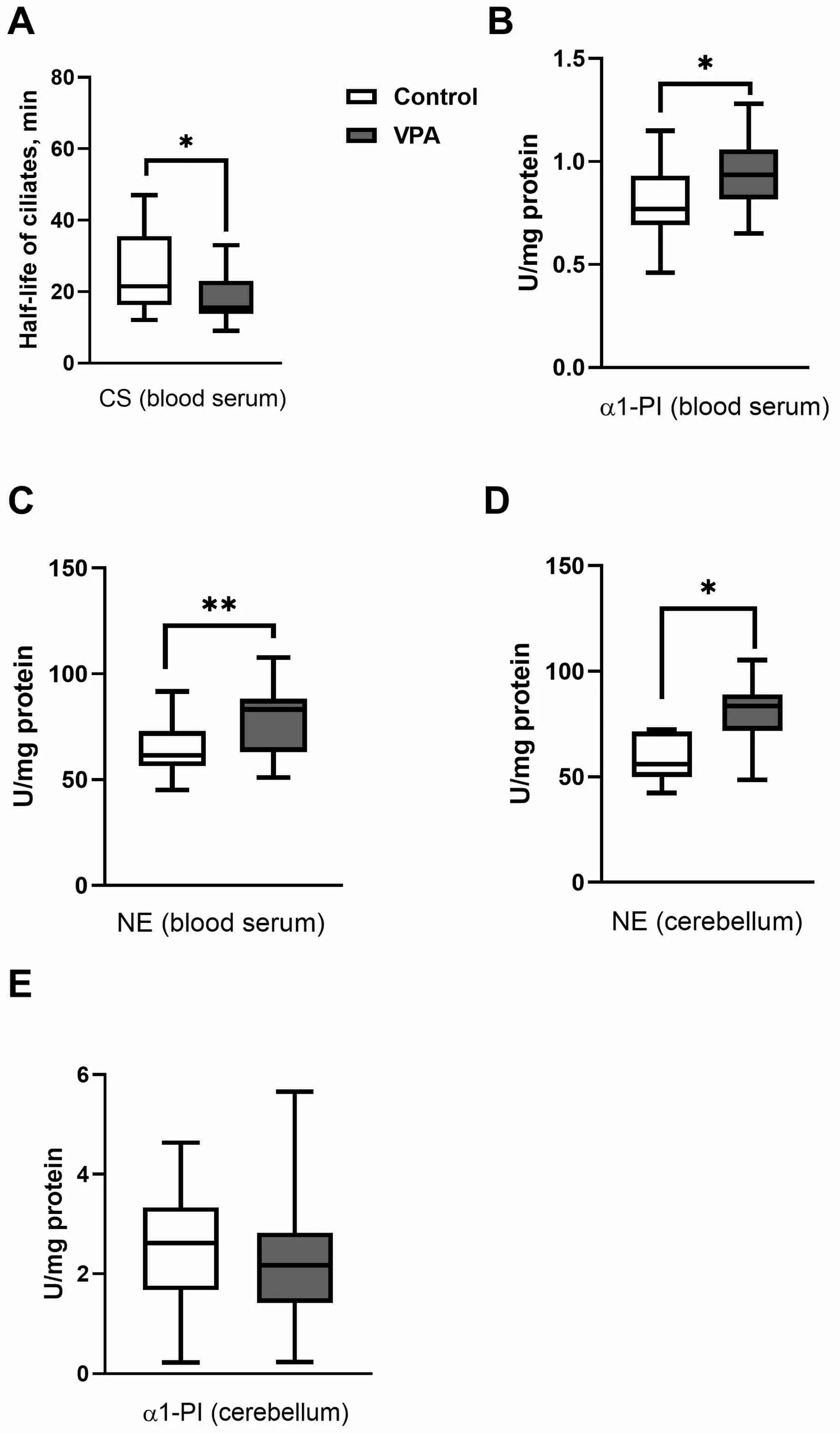
**The effect of postnatal administration of valproic acid (VPA) on the activity of inflammatory factors in the blood serum and cerebellum of rats.** (**A**) The functional activity of complement system (CS) was determined by the rate of ciliate *Tetrahymena pyriformis* death in the presence of blood serum. CS activity is inversely proportional to the immobilization time of half (T50) of the initial number of ciliates, which was 10,000–15,000 active cells/mL. The half-life time of ciliates was shorter in the presence of VPA treated rats’ serum than that of control ones (* *p* < 0.05). (**B**) α1-Protease inhibitor (α1-PI) activity in test samples was determined by inhibition of the BAEE-esterase activity of trypsin. α1-PI activity normalized to serum protein concentration in the serum of rats treated with VPA was higher than in the control (* *p* < 0.05). (**C**) The activity of neutrophil elastase (NE) was determined by the rate of hydrolysis of specific substrate (N-t-BOC-Ala-p-NP). NE activity normalized to serum protein concentration in animals treated with VPA was higher as compared to control animals (** *p* < 0.01). (**D**) NE activity in the cerebellum of animals treated with VPA was higher than in the control (* *p* < 0.05). (**E**) There was no difference in α1-PI activity in the cerebellum of rats from two studied groups. Control animals (8 males and 10 females) received intraperitoneal injections of water, while experimental animals (10 males and 10 females) received an aqueous solution of VPA at a dose of 150 mg/kg daily during the period from the 6th to the 12th postnatal days (PND) of life.

An important peripheral marker of inflammation is the functional activity of CS. In this study, it was determined by the rate of ciliate death upon addition of animal blood serum. The half-life of ciliates in the presence of blood serum from animals treated with VPA was shorter than that observed when the serum of control animals was added to the medium (Figure 3A). CS activation by VPA indicates the development of an inflammatory process. It should be noted that in the control group, the latent period of paw licking in the Hot Plate test correlated with the half-life of ciliates in the presence of rat blood serum (R = −0.6, *p* = 0.013). However, the inverse correlation between pain sensitivity and CS functional activity was disrupted after the administration of VPA.

Another marker of inflammation is α1-PI. Its activity in the serum of rats treated with VPA was higher than in the control (Figure 3B) Its activity in the serum of rats treated with VPA was higher than in the control and correlated with the latency of paw licking in the Hot Plate test (R = +0.4, *p* = 0.048).

Inflammatory processes are characterized by the activation of neutrophils, whose azurophilic granules secrete elastase into the extracellular space. Neutrophil elastase (NE) activity is considered as a marker of inflammation. In this study, NE activity in the serum of animals treated with VPA (4.6 ± 0.1 mM/min) was higher than that of control animals (4.3 ± 0.1 mM/min, *p* = 0.04). This difference becomes more significant when NE activity is normalized to serum protein concentration (Figure 3C). The obtained results indicate the development of inflammation in the applied experimental model of ASD.

The activity of inflammatory factors was compared in the serum and cerebellum of rats. No direct correlation was found between the tested immune parameters in the brain and blood of rats in any of the studied groups. However, in the cerebellum of animals treated with VPA, NE activity was higher than in the control (Figure 3D) (72 and 62 μM/min mg protein, respectively, *p* = 0.048). No similar differences were found for α1-PI (Figure 3E). The obtained results suggest that the physiological and behavioral disturbances characteristic of ASD, induced by VPA administration in early postnatal rat development, may be associated with the activation of inflammatory processes in the brain due to neutrophil infiltration. Increased NE activity may be an indicator of these processes.

When assessing inflammation using serum marker activity (CS and α1-PI), it was found that the more active the inflammatory process, the lower the pain sensitivity. Conversely, the latency of paw licking on a hot plate correlates negatively with NE activity in the cerebellum (R = −0.8, *p* = 0.00006). After VPA administration, the latter correlation reverses (R = +0.6, *p* = 0.006), further indicating a disruption of the central mechanism regulating pain sensitivity in ASD.

The role of inflammatory factors in regulating social behavior in animals is evidenced by multiple correlations (Table 1). Thus, in the control group, most social behavior test parameters correlate positively with the activity of inflammatory factors in the rats’ serum. While a positive correlation between social test parameters and α1-PI in the cerebellum persisted, the correlation between NE activity in this brain region and social behavior parameters in control animals was negative. An active inflammatory process, stimulated by early postnatal administration of VPA, destroys this neuroimmune interaction–the correlation links found in the normal state disappear.

## 4. Discussion

Autism spectrum disorder is a group of neurodevelopmental disorders that begin in early childhood. This group of disorders is characterized by impaired social communication, restricted interests, and a tendency toward stereotypical behavior. Despite the complex etiology and heterogeneity of ASD manifestations in humans, the development of biomedical and translational research is impossible without animal modeling, primarily in laboratory rodents. Rodents are widely used in experimental neuroscience due to their structural, biochemical, and functional similarities to humans, as well as the ease of experimental manipulation and genetic modification. Modeling ASD in mice and rats allows us to analyze fundamental mechanisms of pathogenesis, develop new diagnostic protocols, and test corrective and preventive therapies for autism.

Genetic models of autism in mice cover a wide range of mutant lines, each of which is used to study different aspects of ASD pathogenesis and has its own advantages and disadvantages. Combined models that include knockouts of several key genes and exposure to external factors are considered more promising.

Various forms of prenatal stress are also used to model ASD in animals. In particular, a model of maternal immune activation by administering poly(I:C) or lipopolysaccharides to pregnant females mimics the effects of infectious factors on fetal development and leads to autism-like behavioral and brain structure abnormalities in the offspring [47,48,49].

Key pharmacological animal models of ASD include several protocols used to induce autistic phenotypes through specific interventions at critical stages of brain development. Thus, prenatal exposure to ethanol and other neurotoxins can induce behavioral and neuromorphological changes similar to those seen in ASD [50]. Repeated neonatal injections of propionic acid to animals induce motor stereotypies, anxiety, hyperactivity, and changes in the microbiome, metabolism, and neurotransmitter expression characteristic of ASD [51].

There are two main experimental paradigms-the prenatal (intrauterine) and postnatal (postnatal) models, which differ in the time and mechanisms of action of VPA. The prenatal model is based on the administration of VPA to pregnant females (usually rats or mice) on days 12–13 of prenatal development, which corresponds to the period of neurulation in the embryo. During this period, active formation of neural connections, neuronal migration, and the development of the serotonergic, dopaminergic, and glutamatergic systems occur [35,36]. This model produces a more severe, persistent, and complex autistic-like phenotype, similar to the development of fetal valproate syndrome in children whose mothers took VPA as an antiepileptic drug during pregnancy [28]. It is accompanied by pronounced irreversible neuromorphological changes, which makes it difficult to use this model for testing drugs that are promising for the treatment of less severe forms of ASD.

In the postnatal valproate model, VPA is administered to rat pups early in life (days 6–12). Since the development of the nervous system in rats is completed approximately 10–14 days after birth, this stage is equivalent to the late embryonic period of human development. These animals exhibit: impaired social play and novelty preference, increased repetitive behavior, and changes in neuronal activity in limbic structures (prefrontal cortex, hippocampus, amygdala). Unlike the prenatal model, postnatal exposure to VPA does not cause severe morphological brain defects or significant Purkinje cell loss, and is characterized by reversible functional impairments in interstructural brain connections [36,38]. This allows the postnatal model to be considered a variant of partial or “behavioral” autism without structural abnormalities.

This particular model of ASD was used in this study. Its validity is supported by physiological and behavioral observations. The slowdown in weight gain we observed (Figure 1C) is characteristic of rat pups that received VPA [36]. A decrease in pain sensitivity (Figure 1D) is also observed in the VPA model of ASD [40,41,42]. The main behavioral indicator of ASD, social deficit, was demonstrated on the 55th day after birth in the social behavior test (Figure 3B–D). In the preliminary experiments, we also used other behavioral tests, which did not show significant differences compared with the control group. To assess cognitive abilities from 40 to 45 days after birth, the Complex Food Maze test was used, which revealed some statistically insignificant impairments in the speed of learning and the retention of acquired skills in rats treated with VPA. No significant effect of VPA on the behavior of rats in the Open Field test (running, rearing, repetitive actions) was found. Specific tests for anxiety were not conducted in this study. Due to the lack of significant effects, the relative stressfulness of these tests, and concerns about their impact on social behavior, we did not use them in this study. The only result partially indicating the effect of VPA on the affective state was a decrease in grooming in the social behavior test (Figure 3E). This effect cannot be definitively interpreted as an indicator of anxious, depressive behavior or negative emotionality without additional behavioral data. Despite the above limitations the deviations we discovered in the physiological development and behaviors of rats treated with VPA in the early postnatal period are characteristic of ASD, which supports the validity of the experimental model used.

We employed this model to study the neuroinflammatory mechanism of ASD development. The research was based on a comparative analysis of physiological and behavioral disturbances and the activity of inflammatory markers in blood serum and cerebellum of rats treated with VPA.

The cerebellum is a crucial part of the brain for developmental pathology. Its development begins during the embryonic stage, continues in utero, and is not completed after birth, making it vulnerable during the perinatal period [52]. During the prenatal period, the expression of genes associated with autism (e.g., *SHANK3*, *EN2*, *RORA*) increases in the cerebellum, and its damage during this period significantly increases the risk of developing ASD [53,54]. The cerebellum plays a key role in the pathophysiology of core autism symptoms, including motor and sensory impairments, memory and language impairments, and difficulties with emotional processing, executive function, and social interaction. These symptoms may be related to both altered cerebellar structure and function and disrupted connections between the cerebellum and key brain regions implicated in autism pathophysiology, including the prefrontal cortex, amygdala, and hippocampus [55]. In experimental models of ASD and in clinical studies, a decrease in the density and number of Purkinje cells, a decrease in the number of pyramidal neurons, and changes in the number and length of dendrites have been observed in the cerebellum [56,57]. There is a hypothesis that morphological changes in the cerebellum are accompanied by neurophysiological disturbances leading to a change in the excitation/inhibition balance, which may affect the spatiotemporal properties of information processing [58,59]. These changes in the cerebellum are accompanied by dysfunction of the GABAergic system [60], increased oxidative stress [61] and neuroinflammation [62].

The objective of our study was to determine the role of neutrophils in the neuroinflammatory mechanism of ASD development. Neutrophils are an important part of the innate immune system. They comprise 40–70% of white blood cells in mammals. Being the main phagocytes in the bloodstream, neutrophils can migrate intensively to sites of inflammation located in other tissues. The presence of NE in the cerebellum of the rats we studied (Figure 3E) is apparently due to neutrophil migration into the central nervous system.

In addition to their phagocytic functions, neutrophils produce a wide range of cytokines, an imbalance of which is associated with the development of autism-like symptoms. Results of meta-analyses and large clinical immunological studies demonstrate a persistent increase in levels of proinflammatory cytokines IL-1β, IL-6, TNF-α, IFN-γ, IL-17, and several chemokines (IL-8/CXCL8, MCP-1, eotaxin-1) in the peripheral blood of patients with ASD. A number of studies have shown that concentrations of IL-1β and IL 6 positively correlate with the severity of autistic symptoms and behavioral disorders [12,14]. Some authors have found a decrease in the concentration of anti-inflammatory and regulatory cytokines (primarily TGF-β1 and IL-10), with low levels of TGF-β1 being associated with greater severity of social interaction disorders and stereotypy [17]. Expression of the NF-κB1, IL-1β, IL-8, and TNF-α genes is significantly increased in peripheral blood mononuclear cells from patients with ASD. In the central nervous system of children with ASD, activation of microglia and astrocytes is accompanied by increased production of TNF-α, IL-6, IL-1β, as well as changes in the levels of TGF-β1 and other chemokines in the cortex, cerebellum, and hippocampus [15,16]. Variations in cytokine genes (IL-1β, IL-6, TGF-β, IFN-γ) associated with the risk of ASD indicate a genetic determination of the immune system to acquire a proinflammatory phenotype [63].

An important question is the cause of the probable neutrophil migration to the cerebellum, indicating the presence of neuroinflammation in the ASD model used. Marked activation of microglia and astrocytes was demonstrated in the brains of patients with ASD, particularly in the cerebellum [64]. One of the characteristic features of ASD is hyperconnectivity, which is higher in children than in adults, and it is suggested that impaired synaptic pruning by microglia during brain development may be the cause of connectivity disturbances [65]. The process of synapse pruning by microglia is essential for normal brain development [66], and microglia activation, which leads to altered function, may be one of the causes of abnormal pruning at certain stages of brain development. Microglia activation is believed to lead to increased production of proinflammatory and antiinflammatory cytokines and other factors that influence astrocyte and neuronal function, as well as synapse development [67]. VPA is toxic for microglia [68,69], and administration of this compound during brain development can model microglia dysfunction in ASD pathogenesis. Notably, prenatal administration of VPA disrupts gut microbiota balance and gut barrier functionality, resulting in increased circulating lipopolisaccaride (LPS) levels and impaired blood–brain barrier (BBB) functioning [70,71]. This, in turn, causes neuroinflammation and the secretion of chemokines, which stimulate neutrophil infiltration into the parenchyma and their reverse transendothelial migration into the bloodstream [72]. Neutrophil activation may contribute to further disruption of the BBB and promotion of neuroinflammation. Thus, the use of VPA likely models the development of ASD at both the brain and gut levels [73], and the presumed neutrophil infiltration into the brain likely reflects pathogenetic processes in ASD.

Another important participant in innate immunity is the complement system (CS). The CS consists of approximately 60 soluble proteins, membrane expressed receptors and regulators. Activation of the CS leads to a cascade of proteolytic reactions which terminal phase results in formation of the membrane-attack complexes (MAC) that forms large pores in the target membranes of pathogens, damaged host cells or apoptotic cells. That particularly way of CS activity we determined by using ciliates in rat blood serum. It is known three pathways that lead to the MAC formation: alternative, classical and lectin. Also, CS is involved in cellular immune response by the attraction of immune cells to the inflammation site via the cells surface receptors for anaphylatoxins C3a, C5a and protein of opsonization C3b, etc. Such receptors present on monocytes, macrophages, neutrophils, eosinophils, B cells, T cells, and dendritic cells [74]. Implicated complement proteins not only promote chemotaxis of these cells but activates them and stimulate the production of proinflammatory cytokines (IL-1, IL-6, TNF-α) [75]. This way the regulatory function of CS in local inflammation is realized. Under normal physiological conditions the function of the CS is tightly balanced by its inhibitors. For example, sialic acids on the surface of host cells cover up them from complement attack by binding to the FH regulator [74].

In addition to its immunological functions in the periphery, CS is involved in brain homeostasis, neurogenesis, neuronal migration, and synaptic pruning [76]. The last mentioned is essential for normal brain development and functioning [77]. In some patients with ASD and in a number of animal models, microglial activation, changes in its polarization, and impaired synaptic pruning, including in the cerebral cortex and hippocampus, have been shown to be associated with complement-dependent mechanisms. The review [78] highlights the heterogeneity of microglial subpopulations in ASD and their involvement in complement-mediated pruning and formation of pathological networks.

Synaptic pruning is mediated through the classical pathway of CS activation with a C1q protein leading [76]. The complement component C1q initiates the binding of C3 on neurons to complement receptor 3 (CR3) on microglia and affects synapses [15]. In contrast to schizophrenia pathology where is displayed excessive synaptic pruning, is considered that ASD exhibits a pruning deficiency: spine density is higher compared to the control group, and this is most frequently observed in individuals with lower cognitive function [77].

A significant reduction in the C1q protein level was detected in children with ASD [79]. Partial deficiency of the C4b protein which is one of the classical pathway effector components is also associated with autism. Genetic association studies have shown that the null allele of the C4b gene is more common in patients with ASD compared to controls [80]. Also decrease in C4b levels was found in their plasma [22]. The activity of FI and its cofactor FH whose responsible for the cleavage and inactivation of C3b and C4b in negative regulators of the alternative pathway was increased. Meanwhile patients with ASD plasma consisted increased levels of C1q, C3, and C5 [81]. Furthermore, it has been shown that complement C3a plays a critical role in the activation of the brain endothelium and the recruitment of leukocytes (mainly neutrophils) into the brain after local stimulation with LPS, which creates conditions for the subsequent activation of microglia [82]. An experimental study demonstrated that mice deficient in C1q and C3 exhibit an increased number of dendritic spines in the brain [83]. Thus, the peripheral inflammation, changes in the activity of neutrophils and their proteases, as well as complement activation can contribute to: increased deposition of complement components (C1q, C3, C4b) on vulnerable synapses, which increases their “visibility” to microglia and stimulates pruning, leading to a shift in microglia from a homeostatic to a proinflammatory phenotype with increased phagocytic activity [78].

The literature suggests an association of certain CS components with ASD. However, data on the resulting functional activity of the CS in ASD are insufficient. Traditionally hemolysis of sheep or human red blood cells is used to determine CS activity in biological samples in vitro but these tests are species-bound and require additional sensitization of the red blood cells with antibodies. These shortcomings can be overcome by using a different cellular target. In the mid-20th century it was discovered that guinea pig complement can immobilize ciliates without requiring additional cell sensitization [84]. Considering this fact we developed a method of the functional complement activity detection where the *T. pyriformis* ciliates surface is targeted for the MAC formation that makes them to slowdown first then lysis [45]. The specificity of this method is confirmed by the absence of the ciliate’s loss in the medium without Ca^2+^ and Mg^2+^ ions those are necessary for the classical and alternative pathways CS activation. The developed method was applied in a pilot study of the functional activity of the CS in the blood of children with ASD [46].

The result obtained using this method in this work–activation of CS in the blood serum of rats in response to the administration of VPA–indicates the presence of peripheral signs of inflammation (Figure 3A). At the same time, we observed signs of neutrophil activation in the blood of experimental animals and their infiltration into the brain (Figure 3D,E). The interaction of CS with these immune cells may have played a role in these processes. However, unfortunately, testing CS activity in the brain using our method is not possible. Ciliates do not die in brain homogenates. It is possible that some of the proteins from which MAC is assembled are lost during the preparation of the cytosolic fraction of brain tissue homogenate. Besides this, brain homogenate is a good nutrient medium for ciliates, allowing them to reproduce faster than they die, as observed in our experiment. An alternative way to overcome this limitation may be to test individual CS proteins in brain tissue.

Indirect evidence of the involvement of CS in neuroimmune interactions in this study may be the correlation between the half-life of ciliates in the presence of rat blood serum and the latency of their paw licking in the Hot Plate test (R = − 0.6, *p* = 0.013). The role of CS in the regulation of pain sensitivity at the level of the central and peripheral nervous system has been repeatedly described in the literature [85]. The correlation data presented in Table 1 indirectly indicate the positive influence of CS on the development of social skills in animals under normal conditions. It can be assumed that the break in these correlations as a result of the introduction of VPA in the early postnatal period reflects the development of inflammation and the disruption of synaptic pruning characteristic of ASD during the maturation process. Thus, the obtained data indicate that CS, on the one hand, normally mediates neuroprotection and neuroplasticity, and on the other hand, can contribute to the development of CNS pathology through neuroinflammation.

Similar patterns are observed in the correlations between animal social behavior and serum NE and α1-PI activity in control group (Table 1). Apparently, a normally functioning immune system promotes the harmonious function of the central nervous system. The complement system ensures “surgical” precision in pruning unnecessary synapses. A positive correlation between serum NE and social behavior indicates an adequate level of immune protection in these animals. At the same time, the NE/α1-PI system acts as a “protective buffer,” preventing tissue damage from excessive inflammation. On the contrary, high NE activity in the cerebellum is associated with a decrease in social activity in normal animals, as evidenced by the negative correlation between these indicators. This is partly confirmed by increased cerebellar NE and impaired social behavior in rats treated with VPA during maturation. Such correlations, of course, are not proof of the stated assumption, but they provide direction for planning further research.

The results obtained in this study suggest that the physiological and behavioral disturbances characteristic of ASD, induced by VPA administration in the early postnatal period, may be associated with the activation of inflammatory responses in both the blood (Figure 3A–C) and brain (Figure 3D) of experimental rats. Increased NE activity may be an indicator of these processes. This is consistent with our previous study, which reported increased serum NE activity in children with psychotic forms of ASD and its associations with selected clinical measures [27]. Such a correlation may indicate a connection between the activity of NE in the blood and the severity of the pathological process in the brain of these patients.

In the experimental model we used, VPA administration results in damage to the immature brain, where there is a marked vulnerability due to an insufficient supply of antioxidants, and presumably infiltration of activated neutrophils, long-term exposure to which causes behavioral impairments. Similar delayed effects have been shown after traumatic injury to immature brain [86,87]. Neutrophils in the brain could impact the surrounding tissue in multiple ways, one of which is mediated by NE. When released from neutrophils, NE enhances the destruction of the BBB, since it degrades not only elastin, but almost all components of the extracellular matrix (including collagen types I–IV, proteoglycan, fibronectin, and cadherins) [88]. NE is also thought to play a role in exacerbating ongoing inflammation by cleavage cytokines IL-6 and IL-8, key plasma proteins such as immunoglobulin, coagulation and complement factors [89]. Further, by facilitating the conversion of xanthine dehydrogenase to xanthine oxidase, NE activity likely promotes the generation of oxidative stress [90]. The destructive role of NE in the CNS can be confirmed by the fact that NE KO immature mice showed better improvement in long-term behavior after brain ischemia than WT mice [86]. In our study, traumatic effects on the immature brain, specifically early postnatal administration of VPA, resulted in a delayed impairment of social behavior in rats (Figure 2), apparently due to neutrophil infiltration into the brain, as evidenced by an increase in cerebellar NE (Figure 3D). Additionally, the destructive role of brain-penetrating neutrophils in the formation of mental reactions is evidenced by a negative correlation between social activity indicators in control rats and the activity of NE, but not its inhibitor, α1-PI, in the cerebellum (Table 1). The positive correlation observed between NE activity in the cerebellum and pain sensitivity in rats (R = 0.8, *p* = 0.00006) changes to the opposite after the administration of VPA (R = − 0.6, *p* = 0.006), which indirectly confirms the fact that a decrease in pain sensitivity in ASD may be associated with neuroinflammation.

At the same time, no direct correlation was found between NE activity in the cerebellum and blood of rats in any of the studied groups. The general lack of these relationships can be explained by the relative independence of the development of inflammatory processes in the brain and periphery [91]. In addition, it is important to take into account that in the serum we determined the activity of NE that entered the blood as a result of neutrophil degranulation in vivo. NE activity determined in the cerebellar homogenate included both enzymes that were originally in the extracellular environment and those that entered there as a result of the destruction of brain tissue cells and, probably, neutrophils infiltrated there.

Unlike most studies, which assessed NE by the amount of immunoreactive material, this study determined the enzymatic activity of NE in both the serum and brain of rats. Limitations of this method may include the presence of endogenous inhibitors and other enzymes with elastase-like activity in the test samples. The first problem is partially solved by adding acetonitrile to the reaction mixture. The second is due to substrate specificity. Boc-Ala-ONp is a suitable substrate for assessing elastase activity in vitro because it contains an alanine ester bond. Other serine proteases, such as trypsin, chymotrypsin, and subtilisin, prefer aromatic amino acids near the peptide bond. In addition to proteases, this substrate is suitable for carboxylesterases, enzymes responsible for the hydrolysis of ester, amide, and thioester bonds in endogenous and xenobiotic compounds.

Due to its higher substrate specificity, NE is the primary enzyme detected in the blood, where neutrophils are constantly present in large numbers. However, only these cells can be the source of NE in the brain, due to their migration in any tissue. Macrophage elastase (matrix metalloproteinase-12, MMP-12) might also contribute to the elastase activity in the brain determined in our study. MMP-12 is produced mainly by alveolar macrophages [92], but can also be synthesized by activated microglial cells in the CNS [93]. Brain serine proteases are enzymes with elastase-like activity that are synthesized by brain cells. Brain serine protease 1 (BSP1), also known as neuropsin, is expressed in the hippocampus and associated limbic structures [94]. Brain serine protease 2 is expressed in several brain regions, but its functions are less studied than BSP1 [95]. Under physiological conditions, brain serine proteases are involved in structural plasticity associated with learning and memory, regulation of synaptic transmission, control of neuronal excitability, and remodeling of the extracellular matrix [96,97]. In pathology, elastases can contribute to the disruption of the blood–brain barrier, enhance inflammatory processes, participate in secondary damage after stroke or injury, and modulate the development of neurodegenerative processes [98]. So, there are several reasons for the increase NE activity in cerebellum. These include neutrophil infiltration, increased BBB permeability, local proteases activity, or residual intravascular blood. Further studies using vascular perfusion and histological analysis are needed to determine the exact sources of increased NE activity in the cerebellum.

Against this background, serine protease inhibitors may play a positive, neuroprotective role. The main physiological regulator of neutrophil elastase activity is serpin α1 antitrypsin (α1-PI). It is synthesized in the liver, released into the blood in the acute phase of inflammation and provides 90% inhibition of NE in the blood [99]. Increasing α1-PI is aimed at suppressing excessive activity of not only elastase-like enzymes, but also trypsin-like proteinases, such as thrombin and plasmin. α1-PI inhibits the activity of kallikrein, factors Xa and X1a, and affects the complement system, reducing the formation of neutrophil extracellular traps (NETs) and the development of immune and thromboinflammation [100]. In addition to antiproteolytic activity, α1-PI has anti-inflammatory and antiviral properties, as well as antiapoptotic action, which significantly expands its role in health and pathology [101]. Therapeutic use of α1-PI or its recombinant forms reduces the level of neutrophil infiltration and the concentration of proinflammatory cytokines even in the absence of NE, indicating elastase-independent regulatory mechanisms [102].

α1-PI reduces blood–brain barrier permeability, modulates microglial activation, reduces the production of proinflammatory cytokines TNF-α, IL-1β, IL-6 and NO and demonstrates a pronounced neuroprotective effect in experimental models of neuroinflammation and injury in the immature brain [103]. On the contrary, a deficiency of α1-PI, caused by a disruption of its synthesis in hepatocytes and a decrease in secretion into the blood, leads to progressive tissue damage and the formation of systemic inflammation [104]. Clinical studies show that increased NE activity with relative α1-PI deficiency correlates with the severity of destructive changes, the risk of complications and an unfavorable outcome [105]. Thus, in children with ASD and their relatives, a reduced level of α1-PI in the blood serum is associated with an increased frequency of occurrence of “deficient” alleles of the α1-PI gene, which is consistent with the hypothesis of a genetically determined disturbance of the protease-antiprotease balance in autism [106].

In our study, increased NE activity was observed in both the serum and cerebellum of rats treated with VPA, which could indicate neuroinflammation maybe because of neutrophil infiltration of the brain. At the same time, an increase in α1-PI activity was detected only in the blood serum of animals in the experimental group (Figure 3B,C). As an acute phase marker, serum α1-PI demonstrated the presence of inflammation. It could be speculated that the lack of growth of α1-PI activity in the CNS complicates its compensatory anti-inflammatory functions, which affects social behavior in VPA-treated animals. Indirect confirmation of the beneficial effect of serine protease inhibitors may be a positive correlation between indicators of social behavior and α1-PI activity in the cerebellum of control rats (Table 1).

Our results show that in healthy (control) rats, significant correlations are found between social behavioral measures and the activity of the complement system (in serum) and neutrophil elastase and its inhibitor (in serum and cerebellar homogenates) (Table 1). This is consistent with the concept of a close interaction between the immune system and behavior, including social behavior. In particular, it was demonstrated that produced by meningeal T cells IFN-γ is necessary for normal social behavior in mice. IFN-γ deficiency or impaired signaling in prefrontal cortex neurons causes deficit in social behavior [107]. It is suggested that even in the absence of infection, in higher species, IFN-γ production by meningeal T cells is enhanced by increased aggregation of organisms, providing a more effective antipathogenic effect [107]. These data suggest that, under normal conditions, the peripheral immune system regulates or even controls social behavior, and, conversely, that social behavior is a regulator of the immune system. It is tempting to speculate that the found associations between social behavior in healthy rats and the activity of a number of immune system components reflect a complex adaptive interaction between the immune system and social behavior. In VPA-treated animals, correlations between these immune parameters and social behavior were not observed. The absence of such correlations may be explained by the suggestion that VPA-induced brain developmental impairments and subsequent social behavior deficit, along with the presence of inflammation and neuroinflammation, disrupt normal regulatory interaction between the immune system and social behavior.

Thus, this study revealed a delayed complex effect of early postnatal VPA administration on the nervous and immune systems of rats in young adulthood. The mechanism of action of VPA on the central nervous system is believed to include toxic effects [68,69] and inhibition of histone deacetylase, which is involved in altering chromatin structure and thus regulates the expression of various genes [32]. VPA induces decreased expression of the glutamate decarboxylase gene and affects the balance of excitatory/inhibitory processes in the nervous system [33]. It is possible that similar epigenetic mechanisms underlie long-term changes in innate immunity. However, this requires further research.

## 5. Conclusions

Early postnatal administration of VPA to Wistar rats induces physiological (delayed weight gain and decreased pain sensitivity during late neonatal development) and behavioral (impaired social behavior in pre-adulthood) changes characteristic of ASD, confirming the validity of the experimental model used. These changes are accompanied by increased activity of inflammatory factors (complement system, α1-PI, NE) in the rats’ serum, indicating the development of an inflammatory process in response to VPA administration. Increased NE activity is observed not only in the serum but also in the cerebellum of rats exposed to VPA, which could indirectly indicate the development of neuroinflammation. Since NE is the main marker of activated neutrophils, an increase in NE activity in the cerebellum can be tentatively interpreted as a consequence of infiltration of neutrophils into the brain or disruption of the BBB. Based on this assumption, a hypothesis has been put forward regarding the significant role of neutrophils in the central inflammatory mechanism of ASD development. Obviously, further experimental studies are needed to confirm this hypothesis. The presented results are consistent with our previous clinical study showing increased serum NE activity in children with severe forms of ASD [27]. However, the question of whether serum NE can be considered a potential marker of neuroinflammation in ASD requires further research. The origin of NE activity in the rat cerebellum and the presence of neutrophil infiltration into the brain or disruption of the BBB in the experimental model used also require further research.

## Figures and Tables

**Table 1:** Correlations between social behavior indicators and the activity of inflammatory factors in the blood serum and cerebellum of control rats.

Title 1	CS Serum	NE Serum	α1-PI Serum	NE Cerebellum	α1-PI Cerebellum
Distance traveled	−0.66 0.048	+0.6 0.044		−0.7 0.033	+0.6 0.044
Climbing to non-sib	−0.6 0.048	+0.55 0.044		−0.5 0.066	
Grooming near non-sib	−0.6 0.044	+0.75 0.033	+0.5 0.066		+0.65 0.04
Time spend in center		−0.5 0.066	−0.55 0.044		
Time spend near non-sib		+0.5 0.066			
Contacts with sib		+0.7 0.033		−0.6 0.044	+0.7 0.037
Contacts with non-sib	−0.5 0.066	+0.6 0.048		−0.55 0.06	
Total contacts		+0.7 0.033		−0.6 0.044	+0.5 0.066
Contacts + Approaches to non-sib	−0.6 0.06	+0.65 0.044		−0.57 0.066	
Contacts + Approaches to sib	−0.57 0.044	+0.7 0.033		−0.6 0.044	+0.7 0.037
Contacts + Approaches + Climbing to non-sib	−0.6 0.044	+0.7 0.037		−0.6 0.06	
Contacts + Approaches + Climbing to sib	−0.6 0.044	+0.7 0.033		−0.6 0.044	+0.6 0.048
Total climbing	−0.6 0.044	+0.6 0.044		−0.65 0.04	

Note. Data are presented as Spearman’s correlation coefficient (R) and Benjamini–Hochberg *p*-value.

## Data Availability

The data presented in this study are available on request from the corresponding author due to privacy and ethical restrictions.
